# Antiferromagnetic THz-frequency Josephson-like Oscillator Driven by Spin Current

**DOI:** 10.1038/srep43705

**Published:** 2017-03-06

**Authors:** Roman Khymyn, Ivan Lisenkov, Vasyl Tiberkevich, Boris A. Ivanov, Andrei Slavin

**Affiliations:** 1 Department of Physics, Oakland University, 146 Library Drive, Rochester, Michigan, 48309-4479, USA; 2 Kotelnikov Institute of Radio-engineering and Electronics of RAS, 11-7 Mokhovaya street, Moscow, 125009, Russia; 3 Institute of Magnetism, National Academy of Sciences of Ukraine, Kiev, Ukraine; 4 National Taras Shevchenko University of Kiev, 03127, Kiev, Ukraine

## Abstract

The development of compact and tunable room temperature sources of coherent THz-frequency signals would open a way for numerous new applications. The existing approaches to THz-frequency generation based on superconductor Josephson junctions (JJ), free electron lasers, and quantum cascades require cryogenic temperatures or/and complex setups, preventing the miniaturization and wide use of these devices. We demonstrate theoretically that a bi-layer of a heavy metal (Pt) and a bi-axial antiferromagnetic (AFM) dielectric (NiO) can be a source of a coherent THz signal. A spin-current flowing from a DC-current-driven Pt layer and polarized along the hard AFM anisotropy axis excites a non-uniform in time precession of magnetizations sublattices in the AFM, due to the presence of a weak easy-plane AFM anisotropy. The frequency of the AFM oscillations varies in the range of 0.1–2.0 THz with the driving current in the Pt layer from 10^8^ A/cm^2^ to 10^9^ A/cm^2^. The THz-frequency signal from the AFM with the amplitude exceeding 1 V/cm is picked up by the inverse spin-Hall effect in Pt. The operation of a room-temperature AFM THz-frequency oscillator is similar to that of a cryogenic JJ oscillator, with the energy of the easy-plane magnetic anisotropy playing the role of the Josephson energy.

An absence of compact and reliable generators and receivers of coherent signals in the frequency range 0.1–10 THz has been identified as a fundamental physical and technological problem^1^,^2^,^3^. The existing approaches to THz-frequency generation, including superconductor Josephson junctions (JJ)^4^, free electron lasers^5^, and quantum cascades^6^ require complex setups, which limit wide use of these devices. At the same time, it has been demonstrated that ferromagnetic (FM) layered structures driven by a spin-transfer torque (STT) created by a DC spin current^7^,^8^, which compensates magnetic damping, can be used as spin-torque or/and spin-Hall auto-oscillators in the frequency range of 1–30 GHz^9^,^10^,^11^,^12^,^13^,^14^,^15^.

In order to increase the generation frequency it was proposed to use *antiferromagnets* (AFM) rather than FM films as active layers of spintronic auto-oscillators^16^,^17^. Unfortunately, the traditional method of the STT-induced damping compensation in FM does not work for AFM. To compensate damping in a FM, the DC spin current must be polarized parallel to the direction of the static equilibrium magnetization. However, since AFMs have two magnetic sublattices with opposite magnetizations, the STT decreasing the damping in one of the sublattices increases it in the other sublattice, thus resulting in a zero net effect. Fortunately, the presence in an AFM of two magnetic sublattices coupled by a strong exchange interaction qualitatively changes the magnetization dynamics of AFM^18^. In particular, it has been shown, that, in contrast with a FM, the STT acting on an AFM can lead to a dynamic instability in the magnetic sublattice orientation^3^,^16^,^19^,^20^, which results in the rotation of the magnetizations of the AFM sublattices in the plane perpendicular to the direction of polarization of the applied spin current^16^,^19^,^20^. This mechanism has been already used to experimentally switch the orientation of magnetic sublattices in AFM materials^21^,^22^. However, the STT-induced rotation of the magnetic sublattices in an AFM has not been recognized so far as a possible mechanism of realization of THz-frequency AFM oscillators, since in a magnetically compensated AFM the steady rotation of sublattices does not create any AC spin-current.

In this work we demonstrate theoretically that a simple structure consisting of a metallic layer with a strong spin-orbit interaction (such as Pt) and a layer of a bi-axial antiferromagnetic (AFM) dielectric (such as NiO) can be a base of a tunable room-temperature THz-frequency signal generator. A DC spin current flowing from a current-driven Pt layer and polarized along the *hard* anisotropy axis of the adjacent AFM layer can excite a rotation of the AFM sublattice magnetizations^16^,^19^,^20^ that is *non-uniform in time* due to the influence of a weak *easy-plane* AFM anisotropy. This non-uniform rotation results in the THz-frequency spin-pumping back into the Pt layer, creating an AC spin current that can be detected using the inverse spin-Hall effect. The generated signal amplitudes of 1 V/cm in the frequency range of 0.1–2.0 THz of the can be achieved for driving DC current densities of 1 × 10^8^ A/cm^2^ to 1.1 × 10^9^ A/cm^2^, that have been experimentally achieved previously in spin-Hall nano-oscillators^12^,^13^,^14^,^15^.

We also demonstrate, that the equations describing the operation of the proposed room-temperature AFM-based oscillator are mathematically analogous to the ones describing the oscillators based on superconducting Josephson junctions (JJ)^23^, with the energy of the easy-plane magnetic anisotropy playing the role of the Josephson energy. Consequently, a number of effects studied previously in JJ oscillators at cryogenic temperatures can also be observed in the room-temperature AFM oscillators. In particular, the inertial nature of the AFM dynamics^18^ leads to the hysteretic behavior of the AFM oscillator, which, therefore, have two different current thresholds: an “ignition” threshold, which is required to start the generation, and a lower “elimination” threshold which, in our case, is twice less then the “ignition” threshold, defining the minimum current density needed to support the generation, once it has been started.

## Results

**Figure 1:**
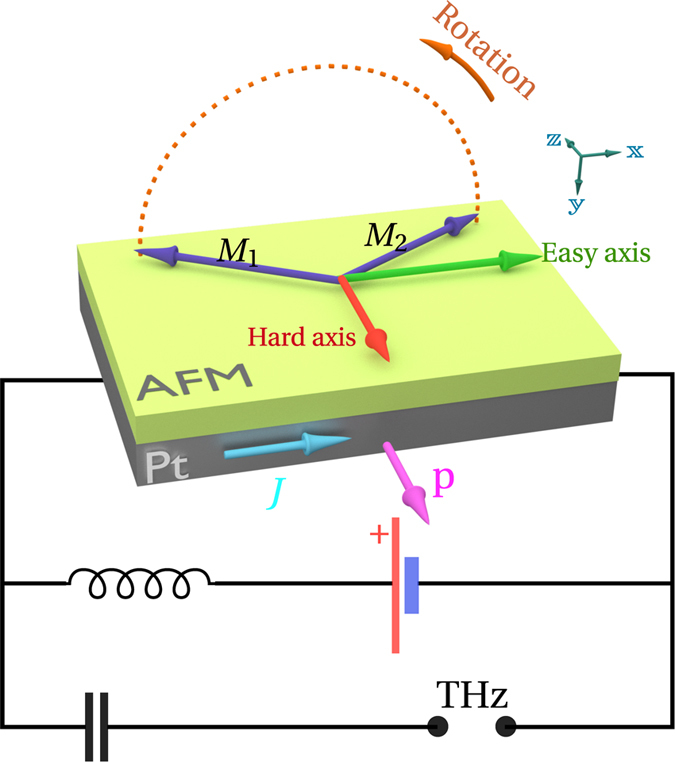
Schematic view of the THz-frequency oscillator based on a Pt/AFM bilayer. The AFM hard axis (HA) lies in the bilayer plane perpendicular to the direction of the DC bias current and parallel to the direction of polarization of the spin-current flowing from the Pt layer into the AFM layer ***p***. Solid dark blue arrows show canted magnetizations under the action of the spin-current. The spin-transfer torque (STT)-induced non-uniform in time rotation of the canted AFM sublattices creates in the Pt layer an AC spin-pumping signal at THz frequencies which is transformed into an AC electric field via the inverse spin-Hall effect in the Pt layer.

We consider a bi-layer consisting of a layer of a heavy metal with strong spin-orbital interaction (e.g., Pt) adjacent to a *bi-axial* AFM layer (e.g., NiO), see Fig. 1. A DC electric current passing through the Pt layer creates, via the spin-Hall effect, a perpendicularly-polarized spin current flowing into the AFM layer^24^,^25^,^26^. Spin current creates a non-conservative spin-transfer torque (STT) on AFM sublattice magnetizations ***M***_*j*_ (*j* = 1, 2): *τ*_STT_ = (*τ/M*_*s*_)***M***_*j*_ × (***M***_*j*_ × ***p***)^7^,^19^,^27^, where ***p*** is the direction of the spin current polarization, *M*_*s*_ = |***M***_*j*_| is the static magnetization of a sublattice, and *τ* is the amplitude of the spin current in the units of frequency^28^. If the spin current is polarized *perpendicularly* to the AFM ground state (along the “hard” AFM axis; here we consider only such a configuration), it tilts the magnetizations from their equilibrium opposite orientation ***M***_1_ = −***M***_2_, which creates a strong effective field ***H*** = *H*_ex_(***M***_1_ + ***M***_2_)/(2*M*_*s*_) (*H*_ex_ ~ 10^3^ T is the exchange field) leading to *uniform rotation* (in the absence of the in-plane anisotropy) of the sublattice magnetizations in the plane perpendicular to the spin-current polarization^16^,^19^,^20^, see Fig. 1.

The rotation of the tilted sublattice magnetizations in an AFM induces the spin current flowing back, from the AFM to the Pt layer, via the spin-pumping mechanism^16^,^28^,^29^:


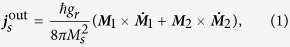


where *g*_*r*_ is the spin-mixing conductance.

The exchange interaction is the strongest interaction in AFM, and, even under the action of an STT, the tilt angle of the sublattice magnetizations is small. Then, introducing the AFM Neel vector^18^,^30^ ***l*** = (***M***_1_ − ***M***_2_)/2*M*_*s*_ the spin-pumping current (1) can be written as:


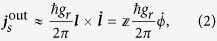


where *ϕ* is the azimuthal angle of the vector ***l***. Equation (2) leads to an important conclusion: *uniform* rotation (with constant angular velocity 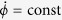) of the Neel vector creates only a DC spin pumping signal in Pt, and creates no AC signal^19^,^20^.

Equation (2) is fully analogous to the second Josephson equation connecting voltage bias in a Josephson junction (JJ) with the phase of the supercurrent^23^,^31^. Similarly to JJ oscillators, to achieve the AC generation we need one more ingredient: a potential “force” that depends on *ϕ*. In Josephson junctions this potential comes from the tunneling Hamiltonian (or Josephson energy)^23^. In AFM, the role of the Josephson energy is played by the energy of crystalline magnetic anisotropy *W*_*a*_ *in the easy plane*: 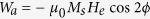, where *H*_*e*_ is the easy-plane anisotropy field.

**Figure 2:**
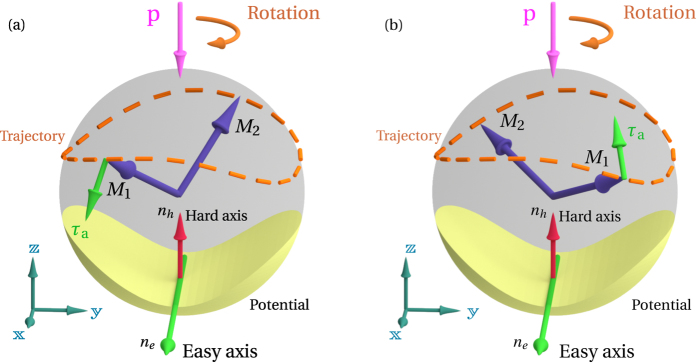
Schematic representation of the rotating sublattice magnetizations in an anisotropic antiferromagnet under the action of an STT. The presence of the easy-plane magnetic anisotropy (yellow-colored potential) in the AFM layer leads to a variable in time rotation speed of the AFM sublattice magnetizations: (**a**) in a part of the trajectory the anisotropy torque decreases the tilt angle between the magnetizations, thus decelerating the rotation; (**b**) in another part of the trajectory the anisotropy increases the tilt and, thus, accelerates the rotation. This nonuniform rotation results in an AC spin-pumping signal in the Pt layer.

The presence of the anisotropy leads to a qualitative change in the dynamics of the magnetic sublattices: it creates an additional conservative torque ***τ***_*a*_(*ϕ*), which depends on the orientation of the magnetic sublattices, see Fig. 2(a,b). Thus the trajectory of each sublattice magnetization is not anymore a planar circle on the sphere’s equator^16^,^17^, but is a more complicated curve (see Fig. 2(a,b)). In one part of the trajectory the anisotropy decreases the tilt angle between the magnetizations ***M***_1_ and ***M***_2_, thus *decelerating* the rotation (Fig. 2(a)), while in another part the anisotropy increases the tilt and, therefore, *accelerates* the rotation (Fig. 2(b)).

At the same time, the trajectory of the Neel vector ***l*** remains a circle even in the presence of easy-plane anisotropy (see Methods for the derivation details), and can be described with one scalar equation for the angle *ϕ*^17^,^19^,^32^:


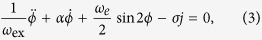


where *ω*_*e*_ = *γH*_*e*_, *γ* is the gyromagnetic ratio, *α* is the effective Gilbert damping parameter, *j* is the electric current density in the Pt layer, and *σ* = *τ/j* is the torque-current proportionally coefficient^16^,^19^. This equation is, essentially, a condition of the balance between four torques acting on the sublattice magnetizations: the exchange torque, created by the exchange field, the Gilbert damping torque, the anisotropy torque, and the STT.

Equation (3) is a well-known equation describing dynamics of a massive particle in a tilted “washboard” potential. It also *exactly* coincides with the equation describing the superconducting phase in a resistively and capacitively shunted JJ under a current bias^33^. Here, the role of the energy stored in a capacitor is played by the exchange energy, which accumulates the kinetic energy in the system, and the role of resistance is played by the Gilbert damping. For sufficiently large currents, equation (3) does not have stationary solutions, meaning that the AFM magnetization sublattices lose their stability, and the Neel vector starts to rotate. The electric current density needed to overcome this “ignition” threshold is proportional to the easy-plane anisotropy:


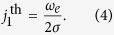


**Figure 3:**
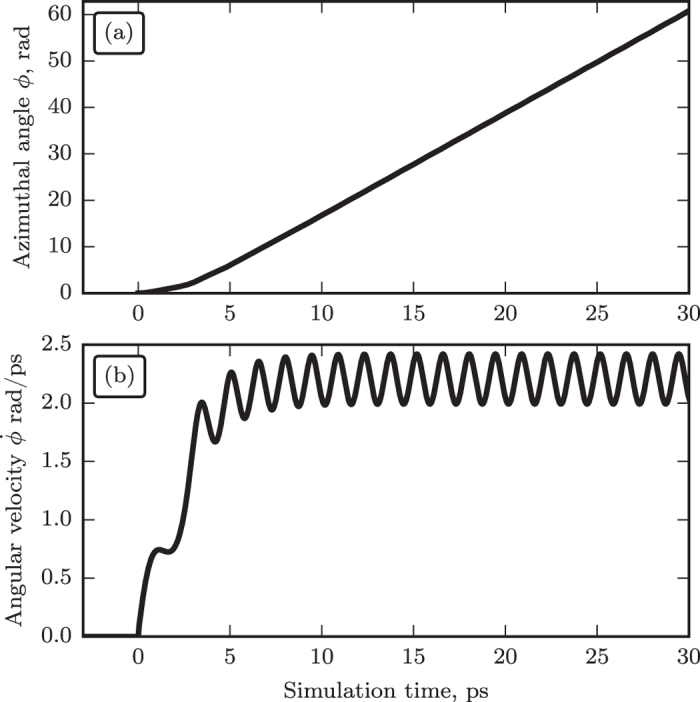
Numerically calculated temporal characteristics of the rotation of the sublattice magnetizations in a bi-axial AFM caused by an abrupt application at *t* = 0 of a supercritical spin current (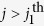 defined by Eq. (4) polarized along the AFM hard axis: (**a**)azimuthal angle *ϕ*; (**b**) angular velocity 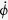 in the AFM easy-plane.

What is more important, the rotation of the Neel vector, due to the influence of the easy-plane magnetic anisotropy, is *not uniform in time*, 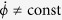. To illustrate this effect, we solved numerically a system of two coupled Landau-Lifshits equations describing the AFM dynamics for a bilayer NiO (5 nm)/Pt (20 nm) (see Methods for other calculation parameters). The time dependence of the azimuthal angle *ϕ* after a sudden application of the DC current is plotted in Fig. 3(a). The angle *ϕ* infinitely increases in time as the system makes revolutions around the direction of the spin current polarization^20^, but its motion *is not uniform in time* due to the action of the easy-plane anisotropy. This non-uniformity is further illustrated by the time dependence of the angular velocity 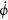, which oscillates in time with THz frequency (see Fig. 3(b)). Thus, the (easy-plane) AFM magnetic anisotropy, existing in the plane perpendicular to the spin current polarization, leads to the generation of an AC output spin-current (2), and turns a current-driven Pt/AFM bi-layer into a potential THz-frequency auto-oscillator.

Another important feature, which is evident from Fig. 3(b), is the existence of a transitional process from the stationary state to a steady oscillatory motion. In contrast to FM materials, the dynamics of AFM is *inertial*^18^,^34^, so that it takes time for the STT to accelerate the sublattice magnetizations, and the magnetic system accumulates some kinetic energy during this transitional process. The inertial nature of the AFM dynamics implies that the AFM oscillator may exhibit a hysteretic behavior, and that two different threshold currents may exist in the AFM oscillator.

**Figure 4:**
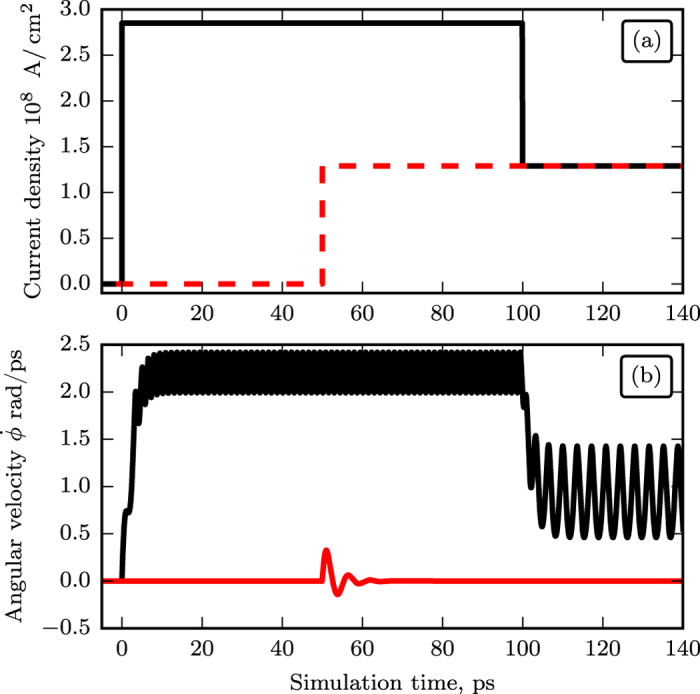
Numerically calculated curves illustrating inertial dynamics of an AFM (NiO) under the action of a DC current step abruptly applied to the adjacent layer of a heavy metal (Pt): (**a**) DC electric current density in the heavy metal layer, (**b**) angular velocity of the Neel vector of the AFM layer. Black curves correspond to the case when the initial magnitude to the DC current density, first, is made higher then the “ignition” threshold (4), and, then, is lowered at *t* = 100 ps to a “working” level. Red curves correspond to the case when the magnitude of the DC current density is abruptly increased just to the “working” level at *t* = 50 ps.

This process is illustrated by Fig. 4, which shows the dynamics of the AFM angle *ϕ* after application of electric current. In the first case (black curves) the current, first, overcomes the “ignition” threshold 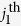, and then is lowered to the “working” density, which is below 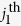. In the second case (red lines) the current is increased from zero directly to the “working” density. In the first case the oscillations continue even after the current was lowered below the “ignition” level, while in the second case the same “working” current cannot start any oscillations. The existence of the hysteresis in the AFM dynamics allows one to “ignite” the generation with a very short (<10 ps) large-amplitude current pulse, and then to reduce the current density for a continuous oscillator operation. The minimum current needed to *sustain* AFM oscillations (the “elimination” threshold) can be found analytically in the limit of a small damping from the condition that the work produced by the STT during one period of rotation equals the energy lost due to the Gilbert damping:


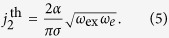


The existence of these two threshold currents is critically important for a practical implementation of the proposed oscillator. For the taken parameters of the bilayer the ignition current density is estimated as 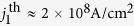, which is larger than the working current for the already demonstrated FM spin-Hall oscillators^13^, however, the elimination threshold current density is twice lower 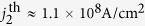, which is *lower*, than the threshold current densities for the FM spin-Hall oscillators. We also note, that similar hysteretic transitions could occur in ferromagnetic spin-torque nano-oscillators, where a spin transfer torque provided by a spin current polarized in a certain particular direction can alter the energy landscape of a magnetic subsystem^35^,^36^.

Equation (3) allows one to find an approximate analytical solution for supercritical currents 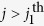:


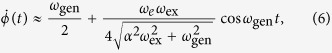


where *ω*_gen_ is the AC generation frequency:


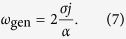


It is clear, that the output AC signal (2), proportional to the variable part of the angular velocity 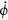, depends on to the anisotropy field *H*_*e*_ in the AFM easy plane, and vanishes for an uniaxial AFM. Also the generation frequency *ω*_gen_ does not depend on the AFM resonance frequencies, but is determined only by the ratio of STT and the Gilbert damping. Similarly, in JJs, the generation frequency depends on current and not on the Josephson plasma frequency.

**Figure 5:**
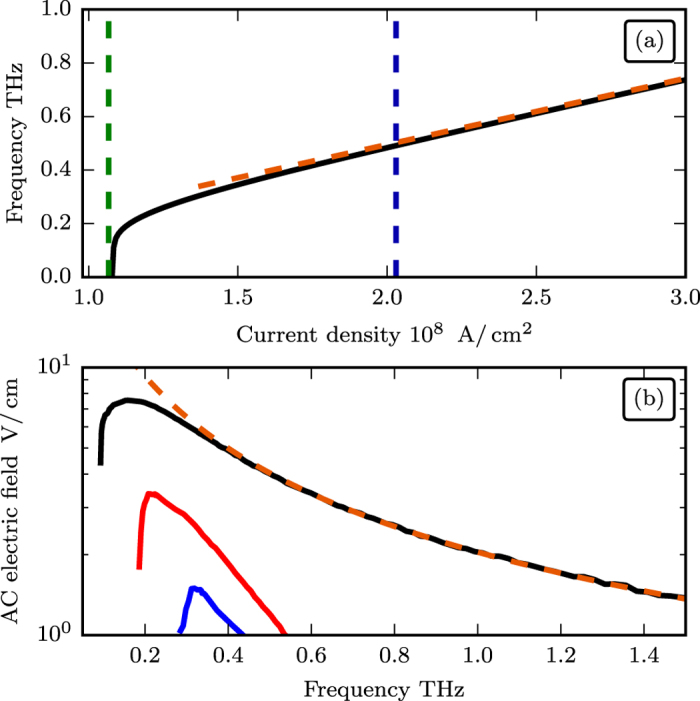
(**a**) Generation frequency *ω*_gen_ as a function of the DC electric current density. Black solid line shows the results of numerical simulations, dashed orange line is obtained from the approximate formula (7), blue and green vertical dashed lines show the “ignition” and “elimination” threshold current densities, respectively. (**b**) Amplitude of the output AC electric field of the AFM oscillator as a function of frequency. Solid lines show the results of numerical simulations (black line – amplitude of the fundamental harmonics *ω*_gen_, red and blue lines – 2nd and 3rd harmonics, respectively). Dashed orange line corresponds to the analytical formula obtained in ref. 29 where the approximate expression for the angular velocity (6) is used. The parameters of the AFM oscillator are given in the Methods.

Figure 5(a) shows the dependence of the generated frequency *ω*_gen_ on the current density in the Pt layer, while Fig. 5(b) – dependence of the amplitudes of several harmonics of the output AC electric field as a function of the generated frequency (see Methods for the parameters of the oscillator). The frequency *ω*_gen_ can be continuously tuned from almost 0 to several THz with current densities *j* ~ 10^8^ A/cm^2^. The first harmonic of the output AC electric field has a maximum at a relatively low frequency ≃0.1 THz, and, then, slowly decreases, remaining sufficiently large (>1 V/cm) up to the frequency of 2 THz. For small frequencies (small currents) the motion of the phase *ϕ(t*) is strongly nonlinear, and the output signal contains multiple higher harmonics, the amplitudes of which decrease rather fast with the increase of the generation frequency.

The linewidth of the generated output signal is an important characteristic of the AFM auto-oscillator (AFMO), determining the spectral purity and the phase noise level of the device. The generation linewidth of an auto-oscillator is mainly determined by the fluctuations of phase of the generated signal^37^. In our model these phase fluctuations were taken into account by adding a stochastic term *f(t*) to the right-hand side of (3). For simplicity, we assumed that the generated frequency *ω*_gen_ is substantially larger than the characteristic frequency of the antiferromagnetic resonance in the used antiferromagnet: 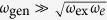. Under this assumption we were able to omit in (3) the term containing the in-plane anisotropy of the AFM, and to solve the obtained stochastic equation:


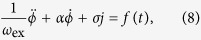


for the case of a random walk of phase^38^. The generation linewidth of the AFMO calculated by the above described method from (8) can be expressed as:


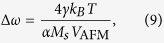


where *V*_AFM_ is the AFM volume, *k*_B_ is the Boltzmann constant and *T* is the absolute temperature of the AFMO. For an AFMO at room temperature and having the lateral dimensions of *L* = 10 μm (see below) the calculated linewidth is Δ*ω*/(2*π*) = 0.36 MHz, which corresponds to the effective quality factor (Q-factor) *Q* ≈ 3 × 10^6^ at the generation frequnecy *ω*_gen_/(2*π*) = 1 THz.

The calculated Q-factor of the AFMO is substantially larger than the quality factor (*Q* = 3 × 10^3^ at the generation frequency of 40 GHz) of a typical spin-torque nano-oscillator (STNO) based on FM materials (see e.g. refs 11,37). The small value of the generation linewidth of the AFMO is related, mainly, to the large energy *E*_0_ of the excited AFM oscillations (*E*_0_/(*k*_B_*T*) = 10^7^ at *ω*_gen_/(2\π) = 1 THz, see Eq. (95) in ref. 37 for details). This is happening for three reasons: (a) the AFMO has a larger volume of the active medium (AFM layer); (b) a maximum 90° precession angle; and (c) an order of magnitude larger precession frequency, compared to a typical FM-based STNO.

There are two other interesting peculiarities of the linewidth expression (9). First, the AFMO linewidth Δ*ω* is independent of the generation frequency (in the limit 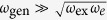), which is caused by the fact, that the average motion and fluctuations of phase, described by the equation (8), are independent. Secondly, contrary to naïve expectations, the linewidth (9) is inversely of proportional to the Gilbert damping constant *α*. Such a behavior of the AFMO linewidth in the limit 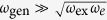, also, can be easily explained. Equation (8) describes a forced drag in a viscous medium, and with a larger damping (viscosity) each random “kick” will lead, on the average, to a smaller deviation of the phase, and, consequently, to a smaller generation linewidth.

## Discussion

Above, we demonstrated that a thin layer of a bi-axial AFM (e.g., NiO) driven by a DC spin current flowing in the adjacent Pt layer can work as a THz-frequency auto-oscillator. Below, we compare the proposed AFMO to other known sources of coherent microwave or THz-frequency radiation.

A working prototype of a spin-Hall nano-oscillator (SHNO) based on a bi-layer of a FM metal and Pt^12^,^13^,^14^ had a threshold current density of *j* ≈ 1.3 × 10^8^ A/cm^2^, which is smaller than the ignition threshold current 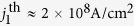, however, this ignition current is needed for only a very short amount of time and can be reduced to lesser current densities above the elimination threshold 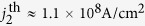, making the proposed AFM/Pt configuration promising for the practical implementation.

The frequency of the SHNO is determined mainly by the bias magnetic field and static magnetization of the FM layer, and was of the order of 5–12 GHz for the bias fields ranging between 400 Oe and 2000 Oe. In the proposed AFMO the generation frequency is determined by the driving electric current, and can be varied from 0.1 THz to 2.5 THz for experimentally achievable current densities (note, that the current densities of up to 1.1 × 10^9^ A/cm^2^ have been achieved in Py/Pt nanowires^14^).

In ref. 17 it was proposed to achieve the THz-frequency generation in NiO/Pt bi-layer via a nonlinear feedback mechanism, which is different from the AFMO generation mechanism described above. In ref. 17 the spin-current is polarized along the *easy* axis of the AFM (easy plane anisotropy field in NiO *H*_*e*_ ≈ 628 Oe^39^,^40^), which leads to a rather large threshold current density, because the current-induced STT has to overcome both the large hard axis anisotropy (hard axis anisotropy field in NiO *H*_*h*_ ≈ 15.7 kOe^39^) and damping. For the parameters used in this work, we estimate this current density (see Methods) as 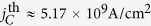, which is more then *H*_*h*_/*H*_*e*_ ≈ 25 times larger than the ignition threshold current for the mechanism proposed here, and, ≈50 times larger then the elimination threshold. Also, the nonlinear feedback mechanism, which stabilizes the AFM precession around the easy axis in ref. 17, works only in a very narrow range of the bias currents and generation frequencies, which severely limits the tunability of the oscillator proposed in ref. 17.

One of the most important characteristics of an oscillator is its output power, which depends on the oscillator’s physical dimensions. The devices working at the frequencies of up to several THz must be smaller than the wavelength *λ*_EM_ of the electromagnetic radiation at these frequencies. To estimate the output power for the above proposed AFMO we assumed here that the working area of the AFMO has the characteristic dimension *L* = 10 μm ≪ *λ*_EM_@1.5 THz ≈ 200 μm. For such a size the output power can be estimated as *W* = *E*^2^*ρ*^−1^*L*^2^*d*_Pt_ (see Methods for parameters). Thus, for the proposed AFMO, the output voltage varies from 6 mV to 1 mV in the frequency range from 0.1 THz to 2.0 THz, which gives the power range from 1.5 μW to 40 nW.

The known oscillators working in the THz-frequency range are the current-biased Josephson junction oscillators working at cryogenic temperatures and having a similar power range^41^, but a much smaller output voltage (16 μV in ref. 42). The amplitude of the output voltage (and, consequently, the output power) of the above proposed AFMO working at room temperature depends on the magnitude of the in-plane anisotropy in the AFM layer (see (6)). To increase the output voltage at higher generation frequencies one can use AFM materials with a stronger easy-plane anisotropy, but this, obviously, will lead to a corresponding increase in the “ignition” threshold current density.

In the above presented calculations it was assumed that the driving spin current is polarized exactly along the hard anisotropy axis of the AFM, and it might be rather challenging to achieve in experiment that the AFM hard axis lies exactly in the plane of the interface between AFM and Pt, containing the direction of the spin polarization. A numerical solution of (11), however, reveals, that a finite misalignment angle Ψ ≲ 60° between the direction of the spin-current polarization vector ***p*** and the AFM hard axis ***n***_*h*_ does not destroy the generation effect, but only leads to an increase of the ignition threshold current as 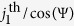. Thus, the proposed THz generation mechanism can be realized even if the AFM hard axis does not exactly coincide with the plane of the AFM/Pt interface.

We also note, that although we took NiO as a model AFM in the above presented calculation, the proposed mechanism of the THz-frequency generation can be realized in other AFM materials, as long as the AFM material has a sufficiently strong easy plane anisotropy (i.e. a definite plane where the vector of antiferromagnetism ***l*** lies), and a weak additional anisotropy inside this easy-plane.

In conclusion, we demonstrated theoretically, that a pure spin current in the AFM layer induced by a DC driving electric current flowing in the adjacent Pt layer can excite THz-frequency oscillations. We showed, that in the case of a NiO (5 nm)- Pt (20 nm) bi-layer it is possible to achieve the generation of 0.1–2.0 THz signals with reasonable current densities that were previously achieved in FM SHNO of a similar geometry. The estimated AC voltage of the proposed AFMO, which can be picked up using the inverse spin-Hall effect in the Pt layer can exceed 1 V/cm for the generation frequencies of up to 2 THz.

## Methods

### Spin-transfer torque

In this work *τ* is the amplitude of the STT expressed in the units of frequency^28^:


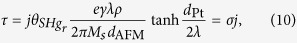


where *j* is the density of the driving DC electric current in the Pt layer, *g*_*r*_ is the spin-mixing conductance at the Pt-AFM interface, *λ* is the spin-diffusion length in the Pt, *ρ* is the Pt electric resistivity, *M*_*s*_ is the saturation magnetization of one of the AFM sublattices, and *d*_AFM_ and *d*_Pt_ are the thicknesses of the AFM and Pt layers, respectively.

### Coupled Landau-Lifshitz equations

Dynamics of a thin antiferromagnetic film can be numerically simulated using two coupled Landau-Lifshitz-Gilbert-Slonczewski equations^7^,^8^,^16^,^43^ for two magnetic sublattices ***M***_1_ and ***M***_2_ of the AFM:


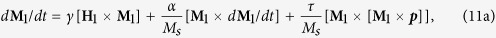



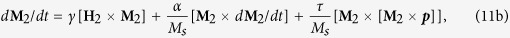


where *α* is the effective Gilbert damping parameter, *τ* is the STT expressed in the frequency units (see equation (10)), ***p*** is a unit vector along the spin current polarization, and **H**_1_ and **H**_2_ are the effective magnetic fields acting on the sublattices **M**_1_ and **M**_2_, respectively:


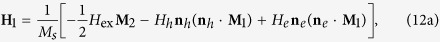



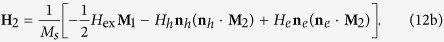


Here *H*_ex_ is the exchange field, *H*_*e*_ and *H*_*h*_ are the easy-plane and hard-axis anisotropy fields, respectively, and ***n***_*e*_ and ***n***_*h*_ are the unit vectors along the hard and easy axes.

### Antiferromagnetic dynamics

To study the dynamics of AFM analytically we use the standard *σ*-model^18^,^30^. The coupled Landau-Lifshits equations (11) can be rewritten in terms of the ***l*** and ***m***. Assuming that |***m***| ≪ |***l***|, which is valid when the exchange field *H*_ex_ is larger than any other field acting in the AFM, one can consider ***m*** as a slave variable:


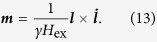


In this approximation the dynamics of ***l*** is governed by one second-order vectorial differential equation:


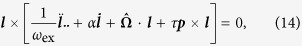


with an additional constraint |***l***| = 1. Here 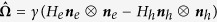, and the symbol ⊗ denotes the direct vector product. Equation (14) is effectively two-dimensional, so we can rewrite it in a spherical coordinate system. To simplify the analytical derivation we assume that 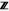 = ***p*** (i.e., the spin current is polarized along the 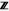-axis), ***n***_*e*_ = 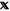, and ***n***_*h*_ = 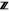:


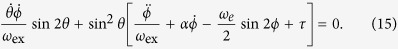



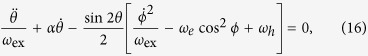


where *ω*_*e*_ = *γH*_*e*_ and *ω*_*h*_ = *γH*_*h*_. The ground state of the AFM corresponds to *θ* = *π*/2 and *ϕ* = 0. The solution *θ* = *π*/2 (i.e., vector ***l*** rotates in the *xy*-plane) is stable for the considered geometry^17^,^19^ and automatically satisfies the equation (16). Using *θ* = *π*/2 in equation (15) it is possible to obtain a single equation (3) for the azimuthal angle *ϕ*.

### Parameters of the system

In all the numerical simulations and estimations reported here we considered a Pt/NiO bilayer with the following parameters: thickness of the NiO and Pt layers *d*_AFM_ = 5 nm and *d*_Pt_ = 20 nm, spin diffusion length in Pt *λ* = 7.3 nm^44^, electrical resistivity in Pt *ρ* = 4.8 × 10^−7^ Ω · m^44^, spin-mixing conductance at the Pt-NiO interface *g*_*r*_ = 6.9 × 10^18^ m^−2 ^32, magnetic saturation of one NiO sublattice *M*_*s*_ = 351 kA/m^40^, spin-Hall angle in Pt *θ*_*SH*_ = 0.1^44^, effective Gilbert damping is *α* = 3.5 × 10^−3^ (see below), exchange frequency *ω*_ex_ = 2*π* × 27.5 THz, *γH*_*e*_ = *ω*_*e*_ = 2*π* × 1.75 GHz and *γH*_*h*_ = *ω*_*h*_ = 2*π* × 43.9 GHz^39^. For the chosen parameters the coefficient *σ* in (10) is *σ*/(2*π*) = 4.32 × 10^−4^ Hzm^2^/A.

### Output electric field

The rotation of the vector ***l*** in the AFM layer induces a spin-current into the adjacent Pt layer, which, in turn, creates an electric field in the Pt layer via the inverse spin-Hall effect (ISHE). This AC electric field serves as the output signal of the AFMO. The ISHE electric field is calculated using the following analytic expression^29^:


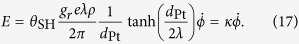


For the chosen parameters of the AFM oscillator the parameter *κ* is equal to *κ* ≈ 1.35 × 10^−9^ V/m ⋅ (rad/s)^−1^.

### Effective Gilbert damping

The intrinsic Gilbert damping constant *α*_0_ for NiO can be calculated from the experimentally measured linewidth Δ*ω*_AFMR_/(2*π*) = 18 GHz of the AFM resonance^39^,^45^. The linewidth Δ*ω*_AFMR_ is related to *α*_0_ by Δ*ω*_AFMR_ = *α*_0_*ω*_ex_, where *ω*_ex_ = *γH*_ex_ = 2*π* ⋅ 27.5 THz is the exchange frequency^40^. One can see, that the intrinsic Gilbert damping in NiO is rather small: 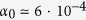. However, the spin pumping from NiO to Pt layer can be described as an additional damping mechanism for the spin dynamics in the AFM^28^,^29^, and the total effective damping constant can be written as:


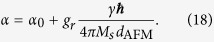


For the chosen parameters of the AFMO the damping parameter is *α* = 3.5 × 10^−3^. Thus, the effective damping in thin Pt/NiO bi-layers is dominated by the spin-pumping mechanism, and strongly depends on the NiO thickness.

### Threshold current in the case when the spin current is polarized along the easy axis

Cheng *et al*. estimated the threshold STT needed to start the oscillations in the case when the spin current is polarized along the easy axis as (see eq. (3) in ref. 17):


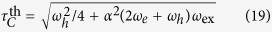


For the parameters of our AFMO, the threshold electric current for the generation mechanism described in ref. 17 can be calculated as:


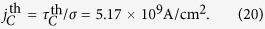


## Additional Information

**How to cite this article**: Khymyn, R. *et al*. Antiferromagnetic THz-frequency Josephson-like Oscillator Driven by Spin Current. *Sci. Rep.* **7**, 43705; doi: 10.1038/srep43705 (2017).

**Publisher's note:** Springer Nature remains neutral with regard to jurisdictional claims in published maps and institutional affiliations.
